# Alcohol reduction strategies among persons with HIV: past attempts, self-reported effectiveness, and future strategies of interest

**DOI:** 10.1186/s13722-025-00581-y

**Published:** 2025-07-20

**Authors:** Nanyangwe Siuluta, Christina E. Parisi, Shantrel S. Candidate, Jacqueline Sherbuk, Yan Wang, Maya Widmeyer, Charurut Somboonwit, Jessy G. Dévieux, Robert L. Cook, Natalie E. Chichetto

**Affiliations:** 1 Department of Epidemiology, University of Florida, 2004 Mowry Road Gainesville, Gainesville, FL 32610 USA; 2 Southern HIV and Alcohol Research Consortium, Emerging Pathogens Institute, Gainesville, FL USA; 3 Department of Psychiatry, Perelman School of Medicine, University of Pennsylvania, Philadelphia, PA USA; 4 Infectious Disease Department, University of South Florida, Tampa, FL USA; 5 Comprehensive Health Care, Melbourne, FL USA; 6 Florida International University, Miami, FL USA

**Keywords:** Alcohol treatment, Patient preference, Treatment effectiveness, People with HIV, Substance use

## Abstract

**Background:**

Alcohol consumption is associated with poor health outcomes in people with HIV (PWH). Although various alcohol reduction strategies exist, little is known about PWH’s past experiences or future preferences. This study describes the previous strategies PWH had used, their perceived effectiveness, among people who ever drank, and the strategies PWH who endorsed heavy drinking would consider trying in the future. It also examines how these experiences and preferences vary by sociodemographic factors and past 12 month drug use.

**Methods:**

A cross-sectional analysis was conducted on data from 453 PWH enrolled in the Florida Cohort Wave III study (2020–2023; mean age 50 years, 60% men). Participants who attempted to reduce or quit drinking (*n* = 321) were asked about their use of eight alcohol reduction strategies and rated the effectiveness of each on a 4-point Likert scale. Participants reporting heavy drinking (*n* = 170) were asked about their willingness to try seven strategies in the future. Chi-square and Fisher’s exact tests analyzed differences by sex, age, race/ethnicity, and past 12 month drug use.

**Results:**

Among the 321 who had ever tried to reduce or quit drinking, endorsed strategies including “on my own”/ complete cessation (80%), prayer (61%), Alcoholics Anonymous (AA) (38%), counseling/therapy (31%), inpatient/outpatient detox (23%), self-monitoring (11%), and medication (7%). The strategies with the highest self-reported effectiveness were for prayer (59%), “on my own”/ complete cessation (58%), and in-patient detox (50%). Prayer was significantly more common among females and non-Hispanic Black or Hispanic participants. Those with past 12 month drug use were significantly more likely to have tried most strategies, except medications or prayer. Among 170 who reported heavy drinking, “on my own”/ complete cessation (43%), AA (24%), and counseling/therapy (21%) were the most endorsed strategies they would try in the future. No significant differences in future preferences were found by demographics, but those with past 12 month drug use showed more interest in formal treatment approaches.

**Conclusion:**

Commonly used alcohol reduction strategies among PWH were non-medical, easily accessible, and perceived as very effective. Incorporating safe and effective patient-driven methods into treatment guidelines may improve strategy uptake.

## Background

Alcohol use is attributed to three million deaths annually, ranking as a leading global cause of death [1]. A study reported that 61% of people with HIV (PWH) in the United States (US) had consumed alcohol within the past year, and 14.6% reported engaging in heavy drinking (defined as four or more drinks in a single day for women and five or more for men) at least once in the past 30 days [2–5]. Numerous studies have shown that increased alcohol consumption among PWH is linked to lower adherence to antiretroviral therapy (ART) and negative health outcomes [6–9]. Those with heavy drinking patterns are at higher risk of experiencing negative health outcomes [10], including poor ART adherence [11], elevated HIV RNA viral load, reduced CD4 cell counts, increased susceptibility to opportunistic infections [12], the development of drug-resistant HIV strains [13], and premature mortality [14]. PWH with heavy alcohol use have greater rates of severe pneumonia, hospitalization, cognitive decline, as well as liver and cardiovascular disease [15–19], compared to PWH without heavy alcohol use. Heavy alcohol use has been reported to affect health outcomes among PWH compared to those who do not consume alcohol or consume it at lower levels [20, 21]. Further, heavy alcohol use is associated with increased mortality rates and reduced life expectancy in this population [22–24]. Therefore, US treatment guidelines underscore the significance of managing heavy alcohol use in HIV care through evidence-based screening and treatment strategies [25, 26].

Research on treatment interventions for those with heavy drinking patterns in primary care settings indicates that brief interventions can effectively reduce alcohol consumption and improve health outcomes [27, 28]. Research on both evidence-based (e.g. Alcoholics Anonymous [AA] and inpatient/outpatient detoxification, screening, brief intervention, and referral to treatment [SBIRT]), and non-conventional treatment (e.g. spirituality), among PWH with AUD is sparse [29–31]. The National Institute of Alcohol Abuse and Alcoholism (NIAAA) has highlighted evidence-based standard treatments for AUD, including behavioral treatments (Motivational Interviewing and Cognitive Behavioral Therapy), support groups (AA), medications (acamprosate, naltrexone, disulfiram), as well as inpatient (IP) and outpatient (OP) detoxification [32]. However, AUD treatment preferences among PWH are unclear, which may affect the uptake of available treatment options if they are not preferred or tailored to their specific needs [33]. Past studies have emphasized the need to incorporate non-conventional strategies such as traditions, religion, spirituality, and cultural heritage in Western approaches to AUD treatment [34, 35]. Studies have reported that prayer is inversely related to alcohol consumption and adopting spiritual and religious lifestyles may increase the likelihood of better outcomes for minority groups with AUD, particularly among Black individuals and Black women [36, 37]. While “complete cessation” (immediate cessation without support) is rarely included as a specific recommendation for alcohol treatment, it has been reported as a popular strategy [38].

Alcohol treatment experiences and future preferences may vary significantly by age, race/ethnicity, sex, and past 12 month drug use. Research indicates that older adults may have different alcohol and drug treatment needs and preferences compared to younger individuals [39], potentially requiring more peer-based support systems. A study by Verissimo and Grella (2017) found that perceived barriers to seeking help for alcohol or drug problems vary significantly by sex and race/ethnicity, with women and racial/ethnic minorities reporting more barriers compared to their male and White counterparts [40]. Notably, a randomized controlled trial on diverse pathways to abstinence among women with AUD found that those who maintained abstinence at treatment entry or planned to quit abruptly (“complete cessation”) had better overall drinking outcomes than those who opted for gradual reduction [41]. Overall, the study emphasized the importance of understanding and addressing the unique pathways that women with AUD take toward abstinence, as well as the need for flexible treatment strategies to accommodate this diversity [41]. Additionally, individuals who currently use drugs may need integrated treatment plans that address both alcohol and past 12 month drug use to achieve better outcomes [42]. Therefore, despite the established efficacy and effectiveness, standard treatments might not account for the preference heterogeneity of individuals with AUD [33].

Therefore, contemporary treatment approaches may need to consider the diverse preferences and heterogeneity of people with HIV and AUD [33, 43]. To our knowledge, the assessment of preferences for different treatments for heavy drinking has not been evaluated among PWH. To bridge this gap, this analysis aims to (1) describe previous strategies used by PWH, (2) their self-reported effectiveness among people who ever drank, and (3) the strategies PWH who endorse heavy drinking would consider trying in the future. We also examined how these experiences and preferences varied by sociodemographic characteristics and past 12 month drug use.

## Methods

### Study design and participants

This study sample was derived from the Florida Cohort Wave III study. The Florida Cohort study is a longitudinal study that investigates healthcare utilization and outcomes among PWH throughout the state to improve healthcare services and overall health.

Participants were recruited from six counties in Florida (Alachua, Brevard, Hillsborough, Marion, Miami-Dade, and Palm Beach) with a strategic over-sample of participants in rural areas [44]. Notably, Hillsborough, Miami-Dade, and Palm Beach are priority areas for the Ending the HIV Epidemic (EHE) Initiative. The EHE initiative is a national effort aiming to reduce new HIV infections in the US by at least 90% by 2030. However, alcohol use is associated with a range of poor HIV-related outcomes, negatively impacting multiple stages of the HIV care continuum—including diagnosis, linkage to care, retention, antiretroviral therapy adherence, and viral suppression [45, 46]. The Florida Cohort study targeted participants mostly from public health organizations to reach a community-based sample that traditionally has limited access to healthcare [45]. Eligible participants included: those diagnosed with HIV, 18 + years old, intended to stay in Florida for at least six months, and could speak in either English, Spanish, or Haitian Creole [36].

**Fig. 1:**
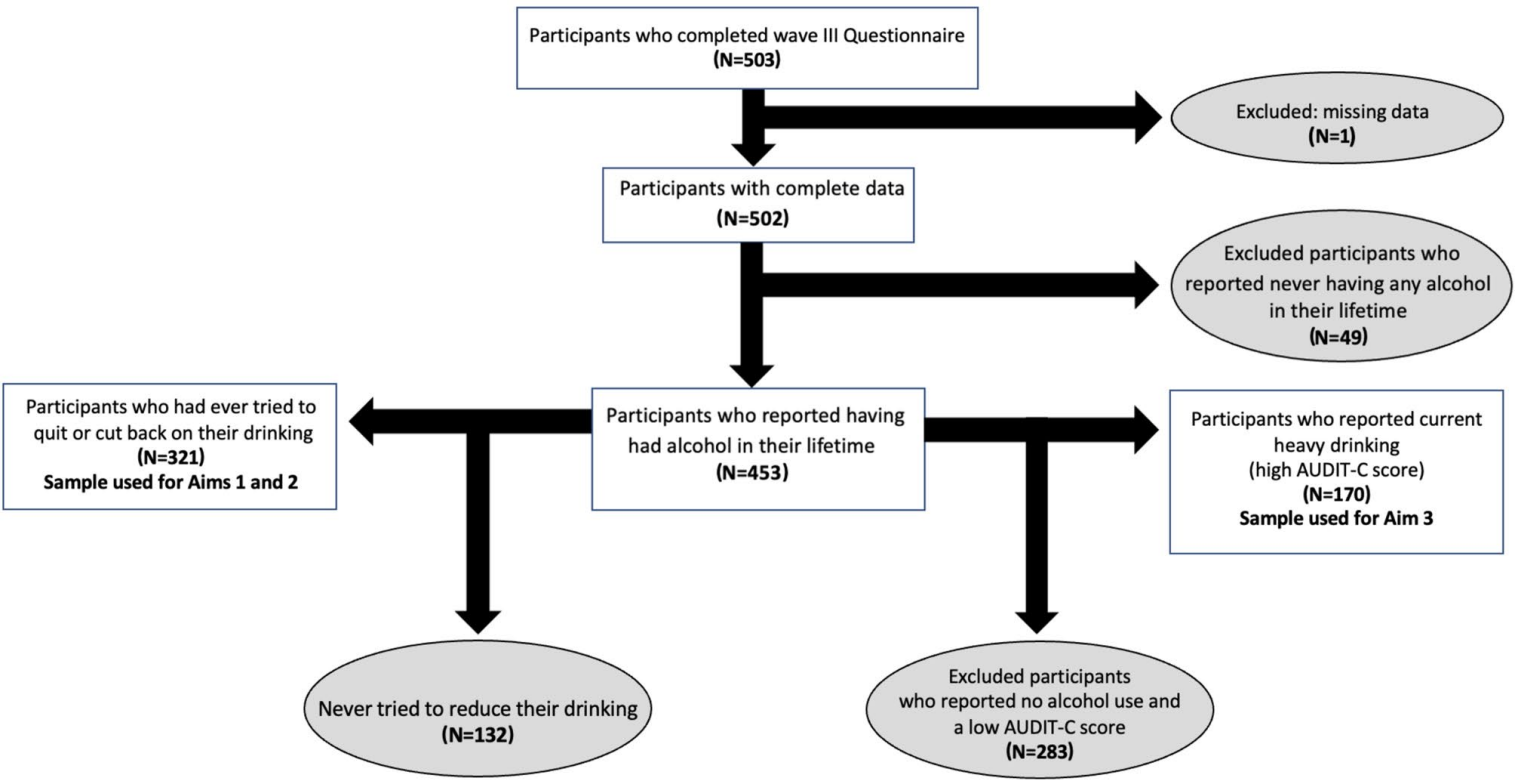
Florida Cohort Wave III participant flow chart

For the current cross-sectional analysis, participants were eligible if they completed the baseline questionnaire from Wave III between October 2020 and February 2023 (*n* = 503) and reported having had any alcohol in their lifetime (*n* = 453). Participants who ever tried to quit or cut back on drinking (*n* = 321) were asked whether they had ever used any alcohol reduction strategies to help them quit or cut back on drinking and participants who reported heavy alcohol consumption (*n* = 170), were asked which strategy they would be willing to try if they were interested in cutting back their drinking (Fig. 1).

### Measures

Self-reported years of age were categorized into three groups (i.e., 18 to 39, 40 to 59 years, and 60 years or older). Employment was categorized as not employed and employed. Race/ethnicity was categorized as Hispanic, non-Hispanic White, non-Hispanic Black, and non-Hispanic multiracial or other race [44]. For this analysis, sex was categorized as male or female. Drug use was evaluated using a list of various substances, including cocaine/crack cocaine, heroin, stimulants including amphetamines, prescription opioids not used under medical supervision, ecstasy/MDMA, hallucinogens, poppers (amyl nitrate), and other drugs. For each specific drug, past 12 month drug use was defined as any drug use (injection or non-injection) within the past 12 months and categorized as Yes/No except for cannabis, which was defined as any use within the previous three months [47].

Alcohol use questions assessed frequency and quantity, and weekly alcohol consumption which was calculated to define Heavy alcohol use. Heavy alcohol use was analyzed using the Alcohol Use Disorders Identification Test (AUDIT-C), a tool designed to detect drinking patterns [4, 48, 49]. In our study, participants assigned female at birth with an AUDIT-C score of 3 or higher, or those assigned male at birth with a score of 4 or higher, were classified as having heavy drinking patterns [4, 50] (Table 1).

**Table 1:** Characteristics of PWH who completed questions on alcohol consumption in the Florida cohort

Variables	Participants who reported ever drinking alcohol (*N* = 453)	Participants who had ever tried to quit or cut back on their drinking (*N* = 321)	Participants who reported current heavy drinking patterns (*N* = 170)
	*N* (%)	*N* (%)	*N* (%)
**Mean Age (Standard Deviation) = 50 (12)**			
**Age**			
18–39 40–59 60+	110 (24%) 230 (51%) 112 (25%)	71 (22%) 176 (55%) 74 (23%)	46 (27%) 83 (49%) 40 (24%)
**Sex**			
Male Female	271 (60%) 182 (40%)	191 (60%) 130 (40%)	97 (57%) 73 (43%)
**Race/ethnicity**			
Hispanic White Black Other	84 (19%) 171 (38%) 187 (41%) 9 (2%)	54 (17%) 122 (38%) 136 (43%) 8 (3%)	31 (18%) 55 (33%) 78 (46%) 4 (2%)
**Past 12 month drug use**			
No Yes	322 (71%) 131 (29%)	223 (70%) 98 (30%)	109 (64%) 61 (36%)

All participants were asked, “Have you ever tried to quit or cut back on your drinking?” Those who responded “Yes” to this question had a follow-up question: “Have you ever tried any of the following treatments or strategies to help you stop or cut back on your drinking?” with the potential answer choices of “Yes” or “No.” Participants were provided with a list of alcohol treatment strategies to endorse any that applied to them: AA; counseling or therapy (counseling/therapy); “detox” or alcohol treatment in an inpatient or hospital setting (IP detox); “detox” or alcohol treatment in an outpatient or community setting (OP detox); medication to help reduce drinking (medication); mobile device, wrist monitor, breathalyzer, etc. (self-monitoring); on my own with no help/“complete cessation” (“on my own”/complete cessation); prayer; and other strategies they could specify in a free-text field. Detox in an IP or OP setting was combined in analyses. When selecting each strategy, participants were also asked a follow-up question, i.e., “How effective was this in helping you stop or cut back on drinking?” To determine the perceived effectiveness of each strategy endorsed by participants, we utilized a 4-point Likert scale, where respondents rated each strategy as either “not at all,” “a little,” “somewhat,” or “very” effective. For the purposes of this study, a strategy was considered to have “perceived effectiveness” if a participant rated it as “very effective.” The proportions of responses indicating a “very effective” rating for each strategy are presented, reflecting the percentage of participants who perceived each strategy as having the highest level of effectiveness.

Participants reporting heavy drinking patterns were asked a follow-up question, i.e., “If you were interested in cutting back on your drinking, which of the following strategies would you be willing to try?” The participants were given the following response options: AA, counseling/therapy; IP detox; OP detox; medication; self-monitoring; “on my own”/ complete cessation; and other strategies they could specify in a free-text field. Notably, prayer was not included as a response option in this set of questions.

### Statistical analysis

We calculated the frequencies and percentages of reduction strategies, perceived effectiveness of strategies, and strategies that they would be willing to use in the future, by sociodemographic characteristics and past 12-month drug use. Bivariate statistics were assessed using chi-square and Fisher’s exact test. The chi-square test (Fisher’s exact test when cell sizes < 5) was used to assess the differences in the prevalence of alcohol reduction strategies used in the past to reduce or cut back drinking (outcome 1) and those willing to be used if interested in cutting back on drinking (outcome 2) by age, sex, race/ethnicity, and past 12-month drug use (exposures). We were also able to detect a large difference with this sample size and p-value < 0.05 and power = 0.08. All statistical analyses were conducted using SAS 9.4.

### Ethics approval

The Institutional Review Board (IRB) at the University of Florida functioned as the official IRB overseeing the Florida Cohort and granted approval for all study materials (IRB#201801680). Prior to participating in the study, all participants provided written informed consent to participate in the Florida Cohort Study.

## Results

Of the total sample of those who ever drank (*n* = 453), 60% were men, 41% non-Hispanic Black, 38% non-Hispanic White, and 19% Hispanic. Over half were middle-aged adults (40–59 years), with a mean age of 50 years (Standard Deviation [SD] = 12 years). Most (71%) reported no past year drug use (Table 1).

### Alcohol reduction strategies used and their perceived effectiveness among PWH who ever tried to quit or cut back on drinking

Of the total sample of those who reported ever using alcohol (*N* = 453), 71% (*n* = 321) participants had reported ever trying to quit or cut back on drinking. Of this subset, the reported strategies used to reduce drinking, were as follows: “on my own”/ complete cessation (80%), prayer (61%), AA (38%), counseling/therapy (31%), IP/OP detox (23%), self-monitoring (11%), and medication (7%). Sex was significantly associated with the use of prayer, which was reported more among women compared to men (72% vs. 53%, *p* < 0.01). Non-Hispanic Black participants were more likely to report using prayer compared with non-Hispanic White participants (76% vs. 41%, *p* < 0.01). Those with past recent drug use reported more use of AA (50% vs. 32%, *p* < 0.01), counseling/therapy (40% vs. 27%, *p* < 0.05), IP/OP detox (31% vs. 20%, *p* < 0.05), and “on my own”/ complete cessation (87% vs. 78%, *p* > 0.05) compared to those without past 12 month drug use (Table 2).

**Table 2:** The percentage of 321 PWH who ever tried each of the alcohol reduction strategies and their associations with socio-demographic characteristics

Characteristics	Reduction Strategies Used in the Past (*N* = 321)						
	AA 121 (38%)	Counseling/ Therapy 100 (31%)	IP/OP Detox 74 (23%)	Medication 24 (7%)	Self-monitoring 34 (11%)	“On My Own”/ complete cessation 258 (80%)	Prayer 195 (61%)
**Age**							
18–39 40–59 60+	29(41%) 69(39%) 23(31%)	25(35%) 55(31%) 20(27%)	15(21%) 42(24%) 17(23%)	7(10%) 11(6%) 6(8%)	9(13%) 20(11%) 5(7%)	59(82%) 142(81%) 57(77%)	41(57%) 104(59%) 50(68%)
**Sex**							
Male Female	76(40%) 45(35%)	60(31%) 40(31%)	42(22%) 32(25%)	13(7%) 11(8%)	19(10%) 15(12%)	159(83%) 99(76%)	101(53%) ** 94(72%) **
**Race/ethnicity**							
Hispanic White Black Other	22(41%) 46(38%) 51(38%) 2(25%)	15(28%) 40(33%) 43(32%) 2(25%)	12(23%) 28(23%) 34(25%) 0(00%)	3(6%) 11(9%) 9(7%) 1(13%)	8(15%) 6(5%) 19(14%) 1(13%)	44(81%) 103(84%) 104(77%) 6(75%)	36(67%) ** 51(41%) ** 104(76%) ** 3(38%) **
**Past 12 month drug use**							
No Yes	72(32%) ** 49(50%) **	61(27%) * 39(40%) *	44(20%) * 30(31%) *	15(7%) 9(9%)	18(8%) * 16(16%) *	173(78%) 85(87%)	142(64%) 53(54%)

*p*-value < 0.01 = **p-value < 0.05= *

Among the strategies perceived as “very effective,” prayer received the highest endorsement (59%), followed by quitting “on my own”/ complete cessation (58%), IP detox (50%), medication (43%), and self-monitoring (31%) (Table 3).

**Table 3:** Perceived effectiveness of each of the alcohol reduction strategies reported by 321 PWH who had ever tried them

Characteristics	Reduction Strategies Used in the Past (*N* = 321)							
	**AA**	**Counseling/** **Therapy**	**Detox**		**Medication**	**Self-monitoring**	**On Own/** **complete cessation**	**Prayer**
**Perceived effectiveness**			**OP Detox**	**IP Detox**				
Not at all A little Somewhat Very	21 (18%) 25 (22%) 27 (23%) 43 (37%)	11 (11%) 22 (23%) 30 (31%) 33 (34%)	8 (16%) 9 (18%) 14 (27%) 20 (39%)	6 (10%) 11 (18%) 14 (23%) 31 (50%)	7 (30%) 4 (17%) 2 (9%) 10 (43%)	4 (13%) 8 (25%) 10 (31%) 10 (31%)	23 (10%) 29 (12%) 50 (21%) 139 (58%)	8 (4%) 26 (14%) 40 (22%) 107 (59%)

### Alcohol reduction strategies PWH were willing to use if they were interested in cutting back their drinking among those who reported heavy drinking

Of the total sample of those who reported ever using alcohol (*N* = 453), 38% (*n* = 170) participants reported heavy drinking. Of this subset, the proportions that indicated a willingness to try the following alcohol reduction strategies in the future were: “on my own”/ complete cessation (43%), AA (24%), counseling/therapy (21%), medication (12%), IP/OP detox (11%), and self-monitoring (11%). Past 12 month drug use was associated with willingness to use AA (34% vs. 17%, *p* < 0.05), counseling/therapy (30% vs. 16%, *p* < 0.05), and IP/OP detox (18% vs. 6%, *p* < 0.05) compared to those without past 12 month drug use, respectively (Table 4).

**Table 4:** The proportion of 170 PWH with current heavy drinking who were willing to use each of the alcohol reduction strategies and their associations with socio-demographic characteristics

Characteristics	Reduction Strategies Willing to be Used (*N* = 170)					
	AA 40 (24%)	Counsel/ Therapy 35 (21%)	IP/OP Detox 18 (11%)	Medication 21 (12%)	Self-monitoring 19 (11%)	“On My Own”/ complete cessation 73 (43%)
**Age**						
18–39 40–59 60+	13 (28%) 19 (23%) 8 (20%)	12 (26%) 16 (19%) 7 (18%)	6 (13%) 7 (8%) 5 (13%)	4 (9%) 11 (13%) 6 (15%)	5 (11%) 12 (14%) 2 (5%)	16 (35%) 40 (48%) 16 (40%)
**Sex**						
Male Female	18 (19%) 22 (30%)	21 (22%) 14 (19%)	11 (11%) 7 (10%)	11 (11%) 10 (14%)	11 (11%) 8 (11%)	47 (48%) 26 (36%)
**Race/ethnicity**						
Hispanic Non-Hispanic White Non-Hispanic Black Other	9 (29%) 9 (16%) 20 (26%) 2 (50%)	7 (23%) 10 (18%) 16 (21%) 2 (50%)	3 (10%) 4 (7%) 10 (13%) 1 (25%)	3 (10%) 5 (9%) 11 (14%) 2 (50%)	2 (6%) 7 (13%) 9 (12%) 1 (25%)	9 (29%) 26 (47%) 35 (45%) 2 (50%)
**Past 12 month drug use**						
No Yes	19 (17%) * 21 (34%) *	17 (16%) * 18 (30%) *	7 (6%) * 11 (18%) *	11 (10%) 10 (16%)	9 (8%) 11 (16%)	49 (45%) 24 (39%)

*p*-value < 0.01 = **p-value < 0.05= *

## Discussion

Our study offers insight into alcohol reduction strategies that have been tried, their perceived effectiveness, and future treatment preferences among PWH who may have less access to non-HIV healthcare services than the general population, and therefore may be more likely to use or prefer health intervention options that are outside of the traditional healthcare system.

A majority of PWH who drank reported having ever tried an alcohol reduction strategy of some kind to quit or cut back on drinking. Of those that endorsed a treatment strategy, “on my own”/ complete cessation (80%) and prayer (60%) were the most common reduction strategies used in the past. Likewise, a greater proportion of those who reported heavy alcohol consumption also endorsed “on my own”/ complete cessation (43%) as a prospective reduction strategy they would be willing to try if they needed to cut back on drinking in the future.

The high endorsement of reducing/quitting drinking “on my own”/ complete cessation among those who ever tried to quit or cut back on drinking is contrary to the findings from a study that reported a small proportion of women opting for this strategy, while also being the most effective method (i.e., women who stopped drinking by complete cessation had the lowest follow-up drinking frequency, compared to other reduction methods) [41]. In this study, women who stopped drinking by complete cessation experienced negative consequences of withdrawal at baseline, but the negative consequences became a motivator prompting actions towards abstaining from alcohol and seeking treatment for AUD [41]. Quitting drinking by complete cessation has been reported to be effective in reducing alcohol-related problems among PWH [51]. Studies have reported that abrupt cessation may be associated with better treatment results as compared to other gradual reduction approaches [38, 41, 52]. However, studies have also reported meaningful improvements in quality of life for both gradual reduction and quitting drinking by complete cessation [53, 54]. We may need to interpret our results with caution, considering that PWH may rely on alcohol use as a coping mechanism for life challenges, and it may be difficult for them to stop drinking abruptly. Thus, depending on whether they have regular or heavy drinking, they may benefit from the gradual reduction of alcohol or drinking in moderation rather than quitting drinking by complete cessation, considering that they may also be at high risk of alcohol withdrawal [41, 55]. Hence, when considering the effectiveness of treatment, the level of alcohol consumption may also be a factor to consider in this population.

In this study we also found that those who endorsed reducing/quitting “on my own”/ complete cessation as a reduction strategy were also more likely to report past 12-month drug use. The strategy of reducing/quitting “on my own” is applicable to alcohol use and other substances [38, 56, 57]. However, reducing/quitting “on my own” could lead to alcohol withdrawal if someone had heavy drinking [55]. Hence, if individuals express interest in quitting on their own, clinicians could offer a range of available treatment options. Additionally, greater education is needed about evidence-based treatments for alcohol and other substanceuse disorders, including the risks of abruptly stopping alcohol use without clinical care for alcohol.

Among those who endorsed past use of prayer (61%) to reduce/quit drinking, approximately 59% perceived prayer as “very effective” (Table 3). Our findings are similar to other studies that have found prayer to be associated with lower odds of AUD and better health outcomes [37]. Additional studies have highlighted the importance of both physical activity and spiritual growth in maintaining sobriety [11, 22, 23]. In our study, Black females were more likely to use prayer compared to White males, respectively. This observed sex and racial difference may be attributed to Black females’ higher levels of religious participation, which may offer them a distinct form of social support compared to Black males or White participants [37].

Our findings add to the existing evidence that prayer might be appropriate as a reduction strategy among vulnerable populations [35]. Moreover, one study included both a longitudinal and an experimental component, with the experimental component finding evidence that an increase in prayer was associated with a decrease in alcohol consumption [36]. Incorporating unconventional strategies like traditions, religion, and cultural heritage into Western approaches for the reduction of alcohol use could better address the needs of diverse populations [58–60]. Past 12 month drug use was associated with the endorsement of IP/OP detox as an alcohol reduction strategy used among PWH who ever tried to quit or cut back on drinking. It was also associated with the endorsement of IP/OP detox as an alcohol reduction strategy that participants were willing to use in the future. Our findings align with prior research indicating that the initiation and utilization of detoxification services tend to be more frequent among individuals with a past 12 month of drug use and prior treatment experience, whether for alcohol or drug detoxification [61]. Considering those with past 12 month drug use may have previously engaged in drug detoxification, people with heavy drinking who also had drug use may express willingness and/or comfort with IP/OP detoxification as a future option for alcohol reduction. Research has shown that completing detoxification reduces the likelihood of readmission for alcohol or opioid detoxification, with successful detoxification being linked to increased social support [62, 63]. Among PWH, social support plays a critical role not only in promoting adherence to HIV treatment but also in enhancing engagement with alcohol reduction strategies. Strong social networks can provide emotional encouragement, reduce stigma-related stress, and reinforce positive health behaviors, ultimately preventing relapse and facilitating long-term recovery from substance use [30]. Therefore, alongside the implementation of various reduction strategies, social support could be essential to prevent relapse and facilitate long-term recovery from substance use [64].

Only 37% of PWH who had ever tried to quit or cut back on drinking reported that AA was very effective (Table 3). PWH with past 12 month drug use were more likely to endorse AA as an alcohol reduction strategy they were willing to use in the future (Table 4). These findings were similar to a study that reported drug dependence diagnosis as a factor that facilitated AA attendance [65, 66]. We found counseling/therapy to be endorsed highly (31%) and very effective (34%) among PWH who had ever tried to quit or cut back on drinking. Notably, research on the use of counseling for alcohol reduction among PWH is limited. However, studies in the general population have indicated that counseling, specifically Cognitive Behavioral Therapy (CBT), is more effective than no treatment, minimal treatment, or general support [67]. When compared to other well-established therapies such as motivational enhancement therapy and contingency management, CBT is equally effective, but not superior [67]. Furthermore, some studies suggest that combining CBT with pharmacotherapy is more effective than standard care with pharmacotherapy alone [68]. Given the complex interplay between alcohol use and HIV management, these findings underscore the need for tailored interventions that integrate alcohol reduction strategies with HIV care [69]. Effective counseling approaches, particularly those addressing both substance use and HIV-related health challenges, may improve overall treatment adherence and health outcomes [70]. Expanding access to evidence-based counseling, particularly CBT, and exploring its integration with pharmacotherapy and social support systems, could enhance alcohol treatment efficacy and support long-term recovery among PWH [70].

All in all, there is a need to expand alcohol treatment and interventions for PWH to help improve outcomes along the HIV care continuum and reduce the impact of chronic diseases such as liver disease. Given that PWH may have limited access to overall healthcare and may be more likely to seek options outside the formal healthcare system (e.g., “on my own”/ complete cessation, prayer), it may be beneficial to offer a range of intervention options for individuals to choose according to their preferences and needs.

Overall, the key strengths of this study include (1) multi-site data across Florida including a large (enough to identify associations), representative sample of PWH in care; (2) the ability to compare different reduction strategies across a range of sociodemographic factors; and (3) the availability of perceptions of the effectiveness of the strategies endorsed.

However, this study has limitations to consider. This was a cross-sectional analysis and causal inferences between sociodemographic characteristics with alcohol use treatment preferences and perceived effectiveness could not be explored. Self-reported alcohol reduction strategies were subject to recall and social desirability bias and were potentially under-reported or over-reported depending on the reduction strategy. For example, prayer may have been overreported because it is generally viewed as a positive behavior. The validity of self-reported reduction strategies (i.e., IP/OP detox; medication) could not be determined, as medical records were not available. Additionally, the strategies endorsed were pre-specified and not generated by the participants themselves; therefore, there may be a general under-report of strategies not provided in the questionnaire options. Related, prayer was a prespecified strategy of past use, but not for future use, and this limited our ability to assess whether prayer would be a highly endorsed strategy for the future reduction of drinking.

## Conclusion

Overall, the study suggests that PWH who ever tried to quit or cut back on their drinking used a variety of alcohol reduction strategies, with reducing/quitting “on my own”/ complete cessation and prayer being the most common strategies. Additionally, AA, IP/OP detox, and counseling/therapy were reported by a considerable number of participants. Prayer, quitting “on my own”/ complete cessation, and IP detox strategies were perceived as “very effective”. The most common strategies used for alcohol reduction were non-medical, easily accessible, and could be done by the participant outside of clinical care. Our findings underscore the importance of considering diverse and individualized approaches to alcohol reduction that consider preferences among this vulnerable population. Thus, more patient-driven, physician-supported methods of alcohol reduction may need to be incorporated into alcohol treatment guidelines and encouraged in clinical care.

## Data Availability

The data supporting the results or analyses presented in the paper can be found at https://www.sharc-research.org. The data are available upon reasonable request.
